# Supplementary material for: The Construction and Exploration of a Comprehensive MicroRNA Centered Regulatory Network in Foxtail Millet (Setaria italica L.)
Source: Front Plant Sci. 2022 May 6;13:848474. doi: 10.3389/fpls.2022.848474 (PMC9121102; doi:10.3389/fpls.2022.848474)
Supplement: Supplementary file 4 [file Data_Sheet_4.zip › Sit-miRN3000_Seita.5G259200.1_1202_TPlot.pdf]

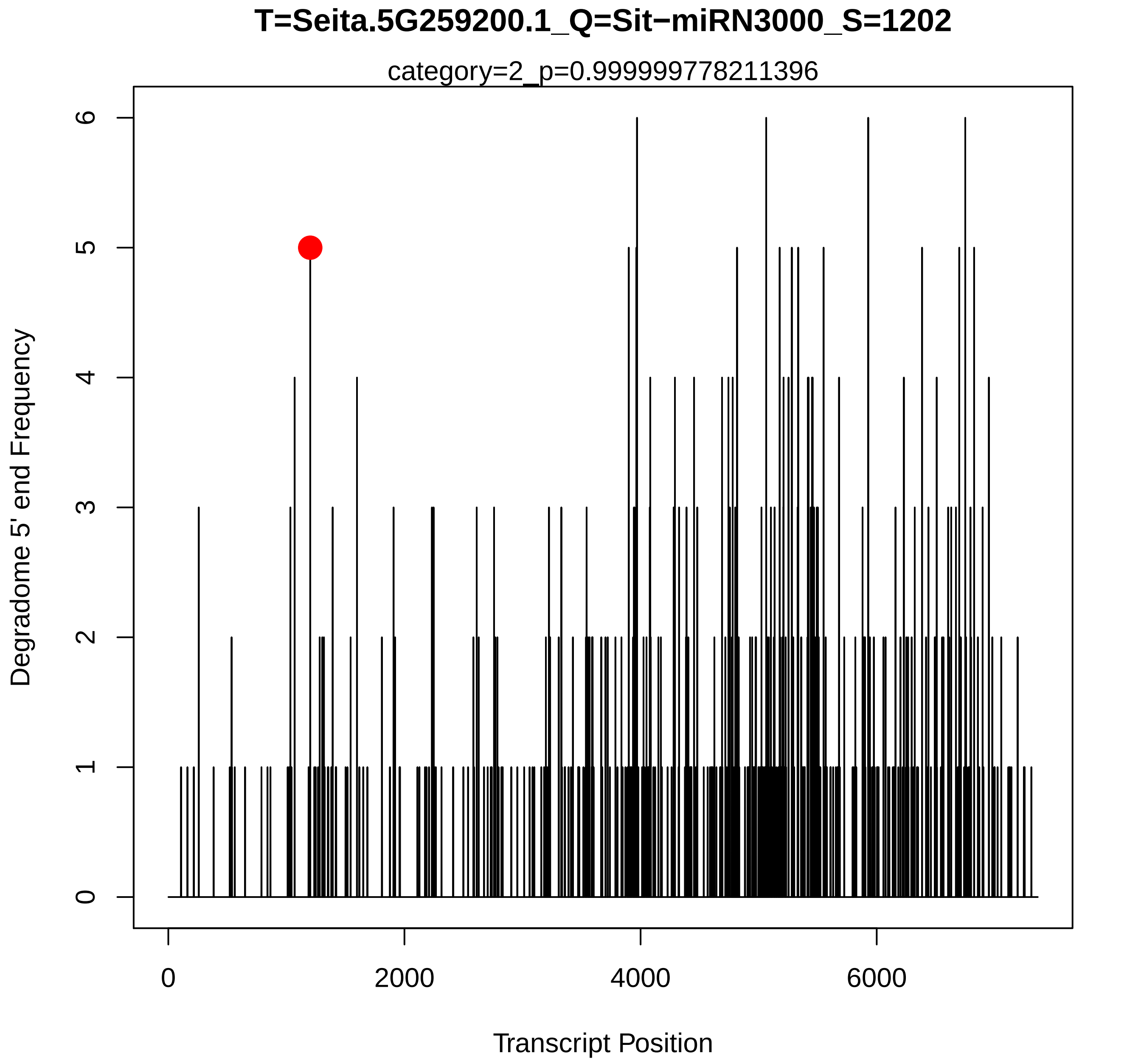
T=Seita.5G259200.1_Q=Sit−miRN3000_S=1202
category=2_p=0.999999778211396
Degradome 5' end Frequency
Transcript Position
0
1
2
3
4
5
6
0
2000
4000
6000
